# Local IL-17A Potentiates Early Neutrophil Recruitment to the Respiratory Tract during Severe RSV Infection

**DOI:** 10.1371/journal.pone.0078461

**Published:** 2013-10-23

**Authors:** Arie Jan Stoppelenburg, Vahid Salimi, Marije Hennus, Maud Plantinga, Ron Huis in ’t Veld, Jona Walk, Jenny Meerding, Frank Coenjaerts, Louis Bont, Marianne Boes

**Affiliations:** 1 Department of Pediatric Immunology and Infectious Diseases, University Medical Center Utrecht, Wilhelmina Children's Hospital, Utrecht, the Netherlands; 2 Department of Virology, Tehran University of Medical Sciences, Tehran, Iran; 3 Department of Pediatric Intensive Care, University Medical Center Utrecht, Wilhelmina Children's Hospital, Utrecht, The Netherlands; 4 Department of Virology, University Medical Center Utrecht, Utrecht, the Netherlands; 5 Department of Pediatrics and Department of Immunology, University Medical Center Utrecht, Utrecht, the Netherlands; University of Cologne, Germany

## Abstract

Respiratory syncytial virus (RSV) bronchiolitis triggers a strong innate immune response characterized by excessive neutrophil infiltration which contributes to RSV induced pathology. The cytokine IL-17A enhances neutrophil infiltration into virus infected lungs. IL-17A is however best known as an effector of adaptive immune responses. The role of IL-17A in early immune modulation in RSV infection is unknown. We aimed to elucidate whether local IL-17A facilitates the innate neutrophil infiltration into RSV infected lungs prior to adaptive immunity. To this end, we studied IL-17A production in newborns that were hospitalized for severe RSV bronchiolitis. In tracheal aspirates we measured IL-17A concentration and neutrophil counts. We utilized cultured human epithelial cells to test if IL-17A regulates RSV infection-induced IL-8 release as mediator of neutrophil recruitment. In mice we investigated the cell types that are responsible for early innate IL-17A production during RSV infection. Using IL-17A neutralizing antibodies we tested if IL-17A is responsible for innate neutrophil infiltration in mice. Our data show that increased IL-17A production in newborn RSV patient lungs correlates with subsequent neutrophil counts recruited to the lungs. IL-17A potentiates RSV-induced production of the neutrophil-attracting chemokine IL-8 by airway epithelial cells in vitro. Various lung-resident lymphocytes produced IL-17A during early RSV infection in Balb/c mice, of which a local population of CD4 T cells stood out as the predominant RSV-induced cell type. By removing IL-17A during early RSV infection in mice we showed that IL-17A is responsible for enhanced innate neutrophil infiltration in vivo. Using patient material, in vitro studies, and an animal model of RSV infection, we thus show that early local IL-17A production in the airways during RSV bronchiolitis facilitates neutrophil recruitment with pathologic consequences to infant lungs.

## Introduction

Respiratory syncytial virus (RSV) infects most infants during their first year of life and is a major cause of hospitalization of otherwise healthy infants in developed countries. The clinical course of severe primary RSV infection is characterized by a marked and rapid neutrophil infiltration, excessive mucus production, and a delayed CD8 T cell response [1,2]. More than 80% of cells in the bronchoalveolar lavage (BAL) in RSV patients are neutrophils [3]. Severity of primary RSV bronchiolitis correlates to the amount of neutrophil infiltration [4], the level of the neutrophil-attracting chemokine IL-8 [5] and, to some extent, the height of the viral load [6,7], which declines prior to the initiation of adaptive immune responses. 

The peak level of neutrophil infiltration into RSV-infected lungs precedes the time point at which the highest viral load is measured in the BAL, and is followed by a compensatory systemic increase of neutrophil precursor cells [2]. Both airway and blood neutrophil subsets were recently demonstrated to support RSV viral replication [8]. Neutrophils contribute to RSV pathology through the production of elastase, which disrupts the lung extracellular matrix and adds to the inflammatory response [4]. The signals that control granulocyte infiltration during acute primary RSV infection are not fully understood.

Single nucleotide polymorphisms in innate immune genes and genes that are involved in the regulation of innate immunity, notably IL-17A, predispose to severe RSV disease [9]. Various lymphoid and myeloid cell types are capable of rapid IL-17A production prior to the induction of adaptive responses [10]. Ever since 2005, an increasing body of literature underlines the importance of IL-17A during the adaptive phase of the immune response to RSV in both mice and men. The role of IL-17A during the innate phase of RSV disease has received little attention. Local IL-17A production depends on anaphylatoxin C3a and tachykinins in RSV infected mice [11]. RSV pathology is enhanced in mice deficient in CCR7 [12] and STAT1 [13] through elevated IL-17A production at 8 days post infection, as well as by Th17 cells that originate from prior sepsis [14]. In its turn, RSV induced IL-17A exacerbates allergic disease in cockroach and ovalbumin models of asthma development in mice, through induction of mucus production and attraction of neutrophils [15,16]. Local IL-17A was previously detected in RSV bronchiolitis patients [15,17] and is elevated during the recovery of infection in infant RSV patients [18]. The relevance and role of IL-17A during the immediate early phase of the RSV immune response, and especially in newborn patients that undergo a primary RSV infection, has remained however unclear. 

We here investigated the possible contribution of IL-17A to lung granulocyte infiltration in human newborns, and the source of IL-17A during the early immune response to RSV infection. Our data show that increased airway IL-17A levels correlate with early neutrophil infiltration into the airways of severe RSV bronchiolitis patients. We clarify the lung resident cell types that produce IL-17A protein early in RSV infection using an RSV infection mouse model. Finally, depletion of IL-17A during early RSV infection in mice reduces neutrophil recruitment to the lungs. Taken together, we propose that local production of IL-17A prior to the initiation of adaptive immunity drives early neutrophil infiltration into RSV infected infant lungs.

## Methods

### Ethics Statement

Ethical approval was obtained for both human and mouse studies. The collection and analysis of human infant tracheal aspirate samples was approved by the local Medical Ethics Committee of the University Medical Center Utrecht (permit number: 09-003), and written informed consent was obtained from the parents or guardians of infant participants.

The mouse study protocols were evaluated and approved by the Committee for Animal Experimentation (DEC) of the University Medical Center Utrecht, Utrecht University, The Netherlands, according to the guidelines provided by the Dutch Animal Protection Act (permit number 2011.II.12.182). Every possible effort was made to minimize suffering of the animals.

### Participants

We collected tracheal aspirate from the ventilation tubes of intubated neonatal RSV infection patients and uninfected infant control patients who were intubated during surgery. Only RSV patients that presented at the pediatric ICU within 6 days of the first disease symptoms were included into this study. We excluded patients with Down syndrome or underlying pulmonary/cardiac complications. 

### Cell lines, antibodies and reagents

A549 cells were purchased from the American Type Cell Culture library (ATCC CCL-185) and maintained sub-confluent in Kaghns-modified F12 medium (Invitrogen) supplemented with 10% fetal bovine serum (FBS), 2mM L-glutamine, 60 mg/ml penicillin, and 20 μg/ml streptomycin. Anti-human antibodies used were purchased from BD Biosciences (Breda, the Netherlands): CD14 (MphiP9), CD16 (3G8) and CD11b (ICRF44). Anti-mouse antibodies for flow cytometry were anti-CD4 (RM  4-5), CD8a (53-6.7), TCR-β (H57-597) (eBioscience (San Diego, CA)); TCR-γδ (UC7-13D5, BioLegend (Uithoorn, the Netherlands)); CD8a (53-6.7), IL-17A (TC11-18H10.1), IL-4 (11B11) (BD Biosciences). Leaf-purified mouse IL-17A neutralizing rat IgG1 (TC11-18H10.1) and corresponding isotype control antibodies were purchased from BioLegend. Recombinant IL-17A was purchased at PeproTech. APC-conjugated PBS-57 loaded CD1d tetramers and A2 strain RSV virus were kindly provided by the National Institutes of Health of the United States of America and Dr. van Bleek, University Medical Center Utrecht, respectively. The RSV stock was grown in HEp-2 cells and precipitated with polyethylene glycol.

### Mice and RSV airway infection experiments

Six to eight week old BALB/c mice were purchased from Charles River (the Netherlands). Mice were intranasally infected with 1 × 10^7^ plaque-forming units RSV/50 μl for high dose infection, and 1 x 10^6^ pfu for low dose infection.

We measured disease progression by daily body weight measurements, which coincides with changes in respiratory function [19]. RSV-infected mice were sacrificed at 2 and 4 days post-infection. Lungs were three times lavaged with ice-cold PBS. BAL fluid from the first lavage was used to determine viral load. Viral loads were measured as described previously [20]. Pooled lavage cells were transferred onto glass slides using cytospin, methanol-fixed and May-Grunwald&Giemsa-stained for light microscopy. Differential cell counts were determined by light microscopy and flow cytometry. Lungs were harvested and digested in RPMI 1640, 10% FBS, 1mg/ml DNAseI, 2.4mg/ml collagenase A.

### RSV infection in epithelial cells

RSV was inactivated by 10 minute UV irradiation. A549 cells were infected with live RSV or UV-inactivated RSV (UV-RSV) at MOI 3 for 24 hours in the presence of 100ng/ml IL-17A. Supernatants were stored at -80 °C prior to Luminex cytokine analysis and cells were lysed in TriPure reagent (Roche Applied Science, Indianapolis, IN) for RNA isolation.

### Tracheal aspirate and cell culture supernatant cytokine analysis

Tracheal aspirates that were used for cytokine analysis were snap-frozen and stored at -80 °C. Several hours before cytokine measurement, we thawed and weighed aspirates and diluted them in HPE buffer (Sanquin). We sonicated aspirates twice at 4°C for 30 seconds, spun down mucus and filtered the supernatant through 0.2µm spin columns (spinX, Corning). Cytokines were measured in the filtered supernatant and results were corrected for dilution. We determined tracheal aspirate and A549 cell culture supernatant cytokine concentrations by multiplex immuno assay as described previously [21].

### Flow cytometry of tracheal aspirate and mouse cells

We harvested tracheal aspirate cells for flow cytometry by vigorous resuspension of aspirates in RPMI, followed by mucus removal by twofold filtration over sterile 70µm cell strainers (BD Biosciences, San Jose, CA). Tracheal aspirate cells were directly surface-stained. 

For intracellular cytokine staining, mouse lung single cell suspensions were stimulated for 4 hours in culture medium with 5 ng/ml PMA, 1μg/ml ionomycin (Calbiochem, Beeston, UK), and 0.66μl/ml GolgiStop (BD biosciences). Stimulated cells were surface-stained in PBS/2%FCS for 30 minutes at 4°C, fixed and permeabilized using a fixation/permeabilization kit for cytoplasmic staining (BD Biosciences), and stained intracellularly in perm/wash buffer for 30 minutes at 4°C. All samples were measured by FACScanto II (BD Biosciences) and analysed using FlowJo software (Tree Star inc.).

### RNA isolation and quantitative PCR

RNA isolation from TriPure was performed according to the manufacturer's protocol. We generated cDNA using a random hexamer primer-based synthesis kit (Fermentas, St. Leon-Rot, Germany). 

Quantitative PCR was performed using the Biorad MyiQV1 and CFX96 systems and miQ SYBR Green ready reaction mix (Biorad). Primer sets used were hIL-8 (Forward: GGCACAAACTTTCAGAGACAG , Reverse: ACACAGAGCTGCAGAAATCAGG), and hGAPDH (Forward: GTCGGAGTCAACGGATT , Reverse: AAGCTTCCCGTTCTCAG). 

### Statistical analyses

Data was analyzed by two-way Student’s T-test and Pearson correlation using Graphpad software. A p-value of at least 0.05 was considered statistically significant. Data are shown as median ± interquartile range.

## Results

### Early increase in tracheal aspirate IL-17A correlates to subsequent neutrophil influx

We asked whether IL-17A contributes to innate neutrophil infiltration in infant RSV patients. IL-17A was earlier related to neutrophil infiltration during established RSV infection in mice [15]. Several studies implicated IL-17A in IL-8 driven neutrophil infiltration to the lungs in other infections [22-25]. 

**Figure 1:**
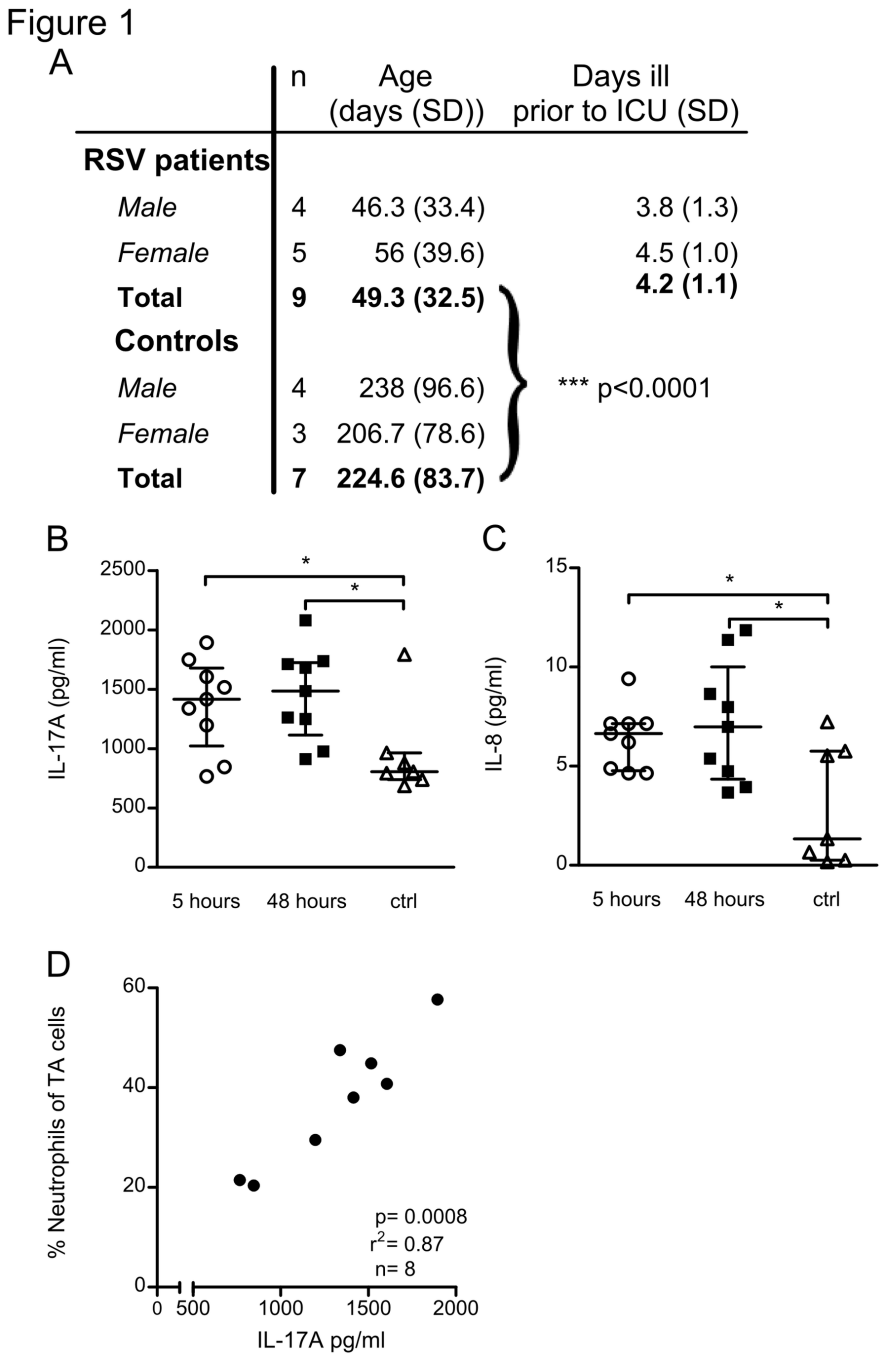
Early tracheal aspirate (TA) IL-17A correlates to subsequent airway neutrophilia. (A) RSV patients in this study were younger than 4 months of age and were included during the innate phase of the immune response. Gender, average age, and duration of RSV disease prior to intubation/inclusion ± SD are shown. (B,C) IL-8 and IL-17A concentrations are elevated in TA of RSV patients compared to uninfected controls. Cytokine levels were determined by multiplex immunoassay from TA that was collected at 5 and 48 hours post-intubation. Data represent 9 patients and 7 controls. (D) TA IL-17A concentration at 5 hours post intubation correlates with neutrophil infiltration at 24 hours post intubation. Neutrophils were detected by flow cytometry and are shown as a percentage of live TA cells (n=8). Each data point represents an individual RSV patient. * denote significance of p<0.05.

Therefore we measured both IL-17A and IL-8 in tracheal aspirates (TA) of infants that had been confirmed to suffer from severe RSV bronchiolitis. RSV infected patients up to the age of 4 months and with less than 7 days of disease history prior to admission to the intensive care unit were included. Otherwise healthy infants that were intubated during surgery served as controls (Figure 1a). We took aspirates for cytokine analysis at 5 and 48 hours after patient arrival and intubation in the pediatric intensive care unit of our hospital. Cellular infiltration was analysed in aspirates that were taken at 24 hours after intubation. Due to technical limitations we could not make simultaneous measurements.

The concentrations of both IL-8 and IL-17A in RSV patient tracheal aspirate were significantly higher compared to uninfected infant controls, and remained stable for up to 48 hours (Figure 1b,c). We next measured neutrophil infiltration in 24-hour TA samples by flow cytometry (Figure S1, gating strategy), and related neutrophil influx to local IL-17A concentrations measured at 5 hours post-intubation. A 24-hour TA sample could not be collected for one patient. Neutrophil influx, expressed as percentage of live TA cells at 24 hour, showed a linear relationship (R^2^= 0.87, p=0.0008, n=8) with the 5-hour TA IL-17A concentration (Figure 1d). Thus, during the initial phase of RSV infection in infant lungs, increased airway IL-17A correlates with subsequent neutrophil recruitment to the lungs. 

### IL-17A and RSV induce increased IL-8 production by airway epithelial cells

IL-17A mobilizes neutrophils to migrate into the lungs through at least two complementary mechanisms: the induction of IL-8 production by epithelial cells [26,27] and induction of adhesion molecules on endothelial cells [28]. Elevated airway IL-8 correlates with the severity of primary RSV infection[5]. Since RSV infection of A549 human airway epithelial cells induces IL-8 production [29], we therefore asked whether IL-17A enhances IL-8 production by RSV infected epithelial cells.

**Figure 2:**
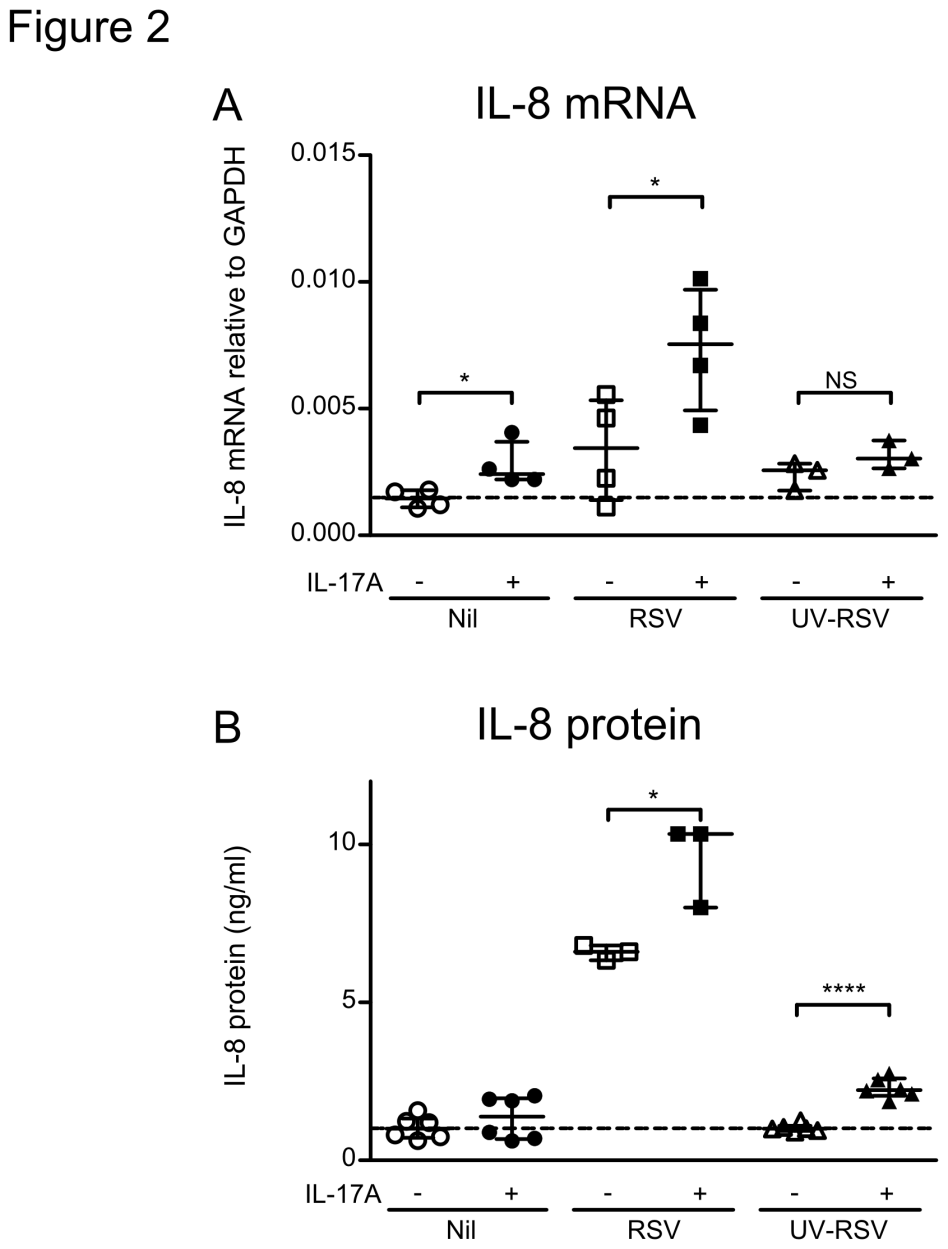
IL-17A and RSV synergistically induce IL-8 production by airway epithelial cells. A549 airway epithelial cells were infected with RSV or UV-inactivated RSV for 24 hours at a multiplicity of infection of 3 and in the presence of IL-17A. (A) IL-8 mRNA levels measured by qPCR. Data represents 3-4 experiments. (B) Concentration of IL-8 in the supernatant of RSV-infected and IL-17A treated A549 cells. Data represents 3-5 experiments. * denote significance of p<0.05, **** denote significance of p<0.0001.

To investigate the combined effect of RSV infection and IL-17A on IL-8 expression, we treated A549 cells with IL-17A and/or RSV for 24 hours and analyzed for IL-8 production at mRNA and protein level by quantitative PCR and cytokine multiplex immunoassay, respectively. Both IL-17A and RSV potentiated IL-8 mRNA levels (figure 2a) and secretion of IL-8 protein (figure 2b), but we observed a synergistic enhancement of IL-8 induction when IL-17A and RSV were administered simultaneously. Using UV-inactivated virus we next tested whether the synergy between RSV and IL-17A in IL-8 induction requires viral replication (Figure 2a,b). While UV-RSV induced similar levels of IL-8 mRNA as infection by (live) RSV in the absence of IL-17A, we observed no synergistic enhancement of IL-8 mRNA expression and a modest, albeit highly significant, enhancement of protein expression when IL-17A was added. Thus, IL-17A requires RSV viral replication for strong augmentation of local IL-8 production in the course of RSV infection.

### High dose RSV infection induces strong early neutrophil infiltration in BALB/c mice

A variety of innate cell types can produce IL-17A during inflammatory conditions, including neutrophils [30], macrophages [31], and early-acting lymphocyte subsets [32-34]. Especially lymphocytes are often located in the lung tissue rather than the lung lumen. Therefore the source of local IL-17A production during early RSV infection cannot be properly investigated in infant RSV patients. Hence we established a mouse model of early primary RSV infection that mimicked neutrophil infiltration in humans. 

**Figure 3:**
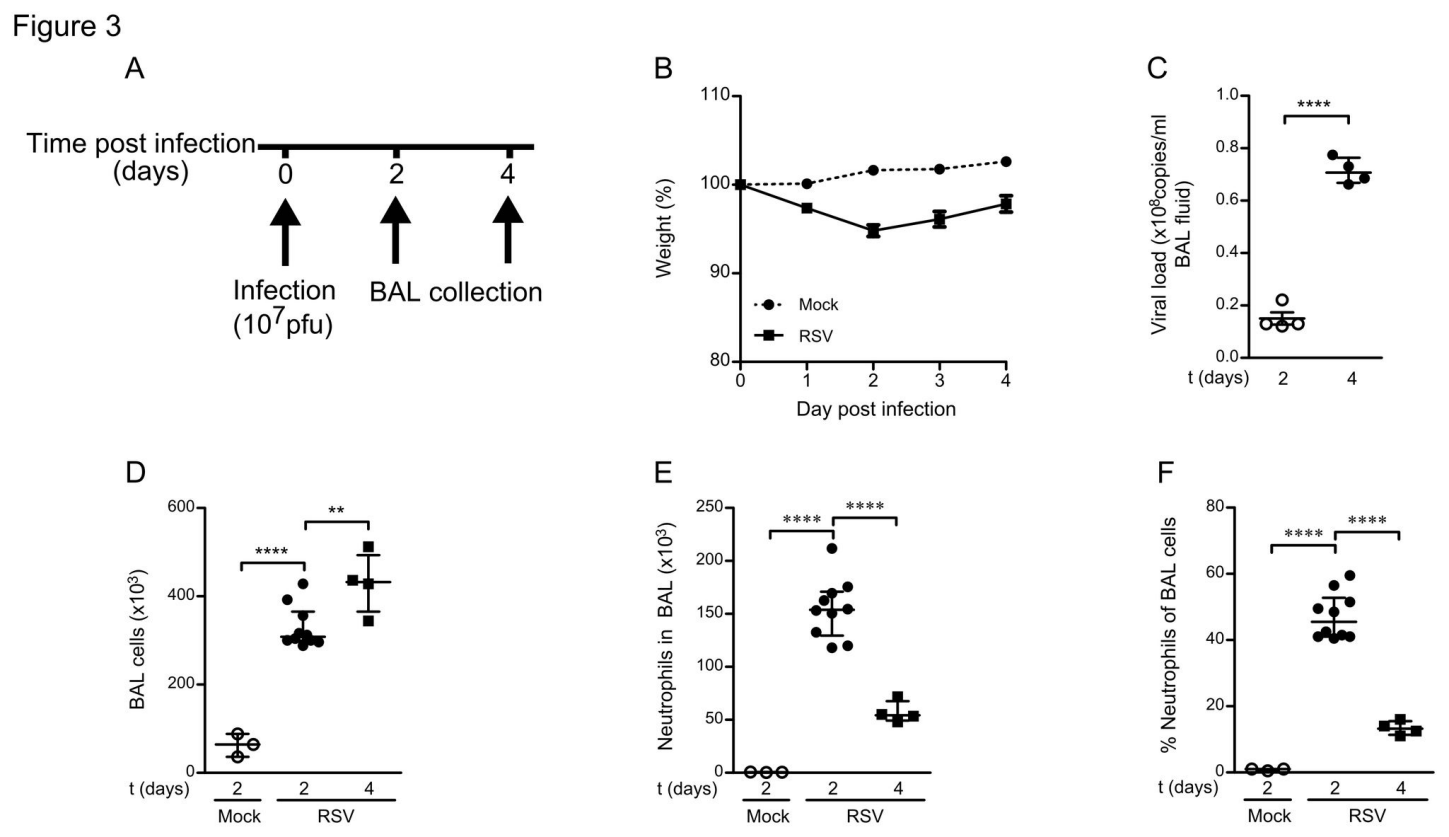
High dose RSV infection induces early neutrophil infiltration in BALB/c mice. (A) Mice were infected intranasally with a high dose RSV (10^7^ pfu/mouse) or mock infected (PBS). BAL was collected at 2 and 4 days post infection. (B) High dose RSV infection induces rapid weight loss in BALB/c mice. Relative weight to the start of infection is shown. (C) RSV replicates in mice. Viral loads were determined by qPCR in the a-cellular fraction of the BAL. (D - F) High dose RSV infection causes infiltration of neutrophils into RSV infected mouse lungs. (D) Live BAL cells were counted using a hematocytometer and trypane blue staining. (E) Absolute numbers and (F) percentages of neutrophils were determined by analysis of May-Grünwald/Giemsa stained cytospins. All data represent 4 - 16 mice per group and three independent experiments. ** denote significance p<0.01, **** denote significance of p<0.0001.

We infected healthy 6-8 week old BALB/c mice intranasally with a high dose RSV (1x10^7^pfu) and followed changes in body weight, viral load, and cellular infiltration until 4 days post infection (Figure 3a, experimental design). RSV infected mice modestly declined in body weight during the first two days of infection compared to mock-infected PBS controls (Figure 3b). The number of viral copies in the BAL fluid increased significantly over time, with peak levels around 4 days post infection (Figure 3c). No virus was detected in mock-infected mice. The amount of immune cells in the BAL fluid accumulated over time and was at all time points significantly greater than in mock-infected mice, where no cellular infiltration was observed (Figure 3d). Neutrophils were the dominant infiltrating cell type at 2 days post-infection, but declined both in proportion and in absolute numbers at 4 days post-infection, as determined by differential counting of May-Grünwald&Giemsa stained BAL cells (Figure 3e, f, and Figure S2). 

As such, we confirmed in our mouse model of RSV infection that neutrophil infiltration precedes the peak level in viral load as in human infant RSV patients. 

### CD4 T cells are major local producers of IL-17A during early RSV infection

In our model of early primary RSV infection, neutrophil infiltration occurred within 48 hours post-infection, effectively ruling out local production of IL-17A that requires adaptive immune processes. Since our patient data underscored a strong correlation of local IL-17A with neutrophil influx, we asked whether other cell types produced IL-17A in BALB/c mice at two days post RSV infection (high dose, 1x10^7^pfu).

**Figure 4:**
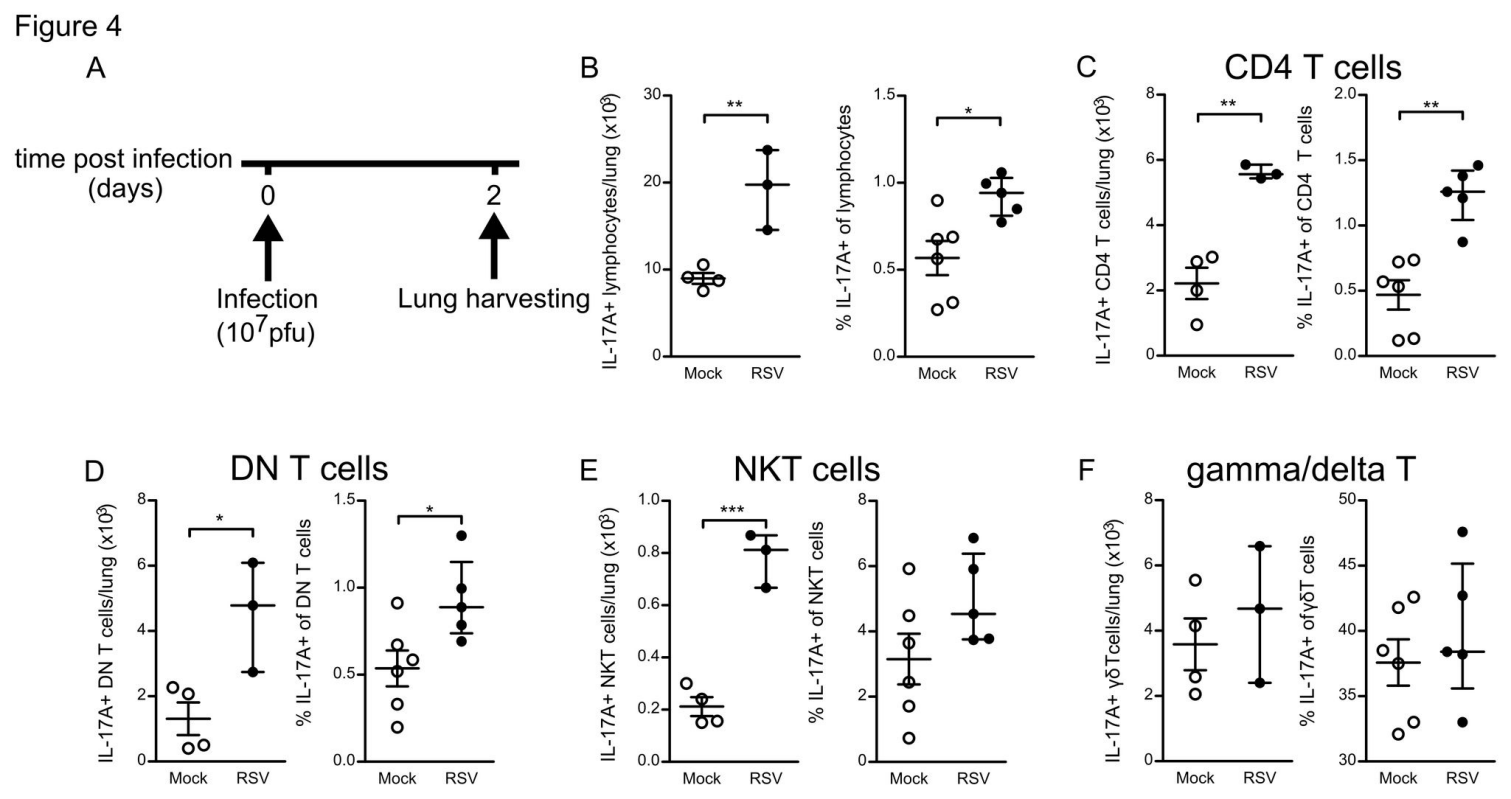
CD4 T cells are major local producers of IL-17A during early RSV infection. RSV induces IL-17A production in lung lymphocytes at 2 days post infection. (A) Lung single cell suspensions were harvested at 2 days post infection and stimulated with PMA/Ionomycin for 4 hours prior to intracellular cytokine staining and analysis by flow cytometry. (B) Absolute and relative numbers of IL-17A producing lung lymphocytes. (C-F) Predominantly CD4 T cells are induced to produce IL-17A by RSV infection. Separate bar graphs of the relative IL-17A production by (C) TCRβ+CD4+ T cells, (D) TCRβ+CD4- CD8- (DN) T cells, (E) CD1d-tetramer+ NKT cells, and (F) TCRγδ+ gamma/delta T cells . Absolute numbers represent n=4 control mice and n=3 RSV infected mice from one representative experiment.* denote significance of p<0.05, ** denote significance of p<0.01, *** denote significance of p<0.001.

To this end we made single cell suspensions of 2 day RSV infected mouse lungs and stimulated them ex-vivo with PMA and Ionomycin in the presence of the Golgi transport inhibitor GolgiStop (Figure 4a, experimental design). Presence of newly produced IL-17A cytokine was measured by flow cytometry of permeabilized cells. We distinguished various cell lineages by surface staining for Gr-1, CD11b, TCR beta, TCR gamma/delta, CD4, CD8, and binding of alphaGalCer-loaded CD1d tetramers. IL-17A production was detected in lymphocytes, but not in cells of the myeloid lineage including neutrophils. Both the absolute number and percentage of IL-17A producing lymphocytes were increased at day 2 of RSV infection, although also mock-infected mice exhibited a considerable fraction of IL-17A producing cells in the lungs (Figure 4b).

The IL-17A producing lymphocyte population consisted of CD4^+^ T cells, CD4^-^ CD8^-^ (DN) T cells, gamma/delta T cells, CD1d/alphaGalCer-tetramer positive NKT cells, and a small number of other lymphocytes including CD8+ T cells and TCR^-^ lymphocytes. Gamma/Delta T cells, CD4 T cells, DN T cells, and to a lesser extent NKT cells were steady state producers of IL-17A in uninfected mouse lungs (Figure 4c-f). 

RSV infection significantly increased the absolute numbers in the lungs of IL-17A producing CD4^+^ T cells, DN T cells, and NKT cells at 2 days post infection (Figure 4c-e). For IL-17A producing gamma/delta T cells, RSV infection did not cause significant changes in cell numbers, although appeared trending towards increased numbers (Figure 4e,f). Within the CD4+ T cell population, we observed a highly significant increase in relative numbers of IL-17A producing cells upon RSV infection (Figure 4c), establishing CD4+ T-cells as major contributors of elevated IL-17A production during early RSV infection. 

These data show that in our model of primary RSV infection various types of lymphocytes, but especially an innate occurring CD4 T cell population, are induced to produce IL-17A at the peak moment of lung neutrophilia. 

### Depletion of IL-17A limits neutrophil infiltration of RSV-infected lungs

**Figure 5:**
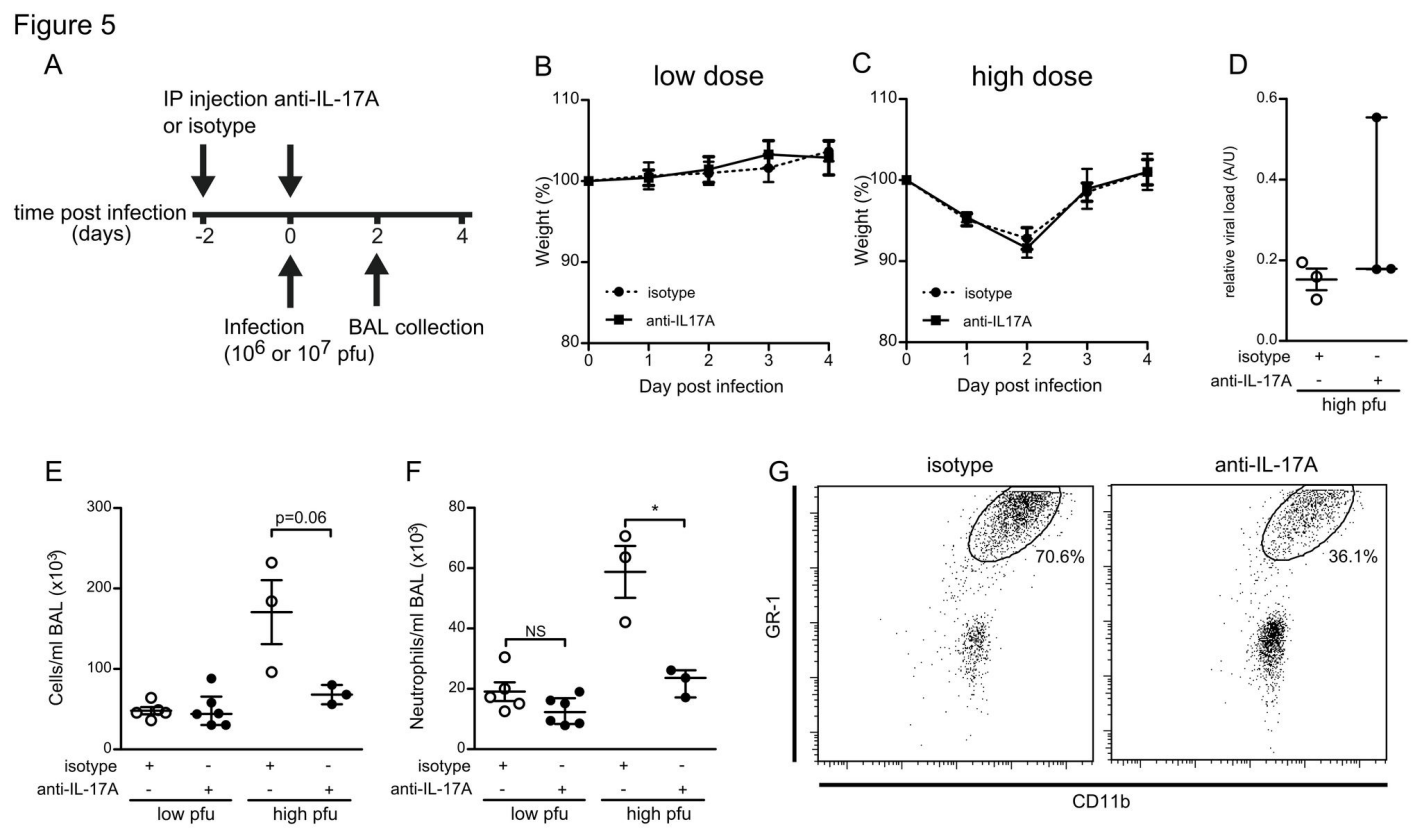
Depletion of IL-17A limits neutrophil infiltration into RSV-infected lungs. (A) Mice received IP injections of anti-IL-17A antibody or isotype control antibody upon and two days prior to RSV infection. Mice were infected with high or low dose RSV and sacrificed 2 days post infection. (B,C) Graphs representing the weight loss of anti-IL-17A treated versus isotype treated mice that were infected with a low or a high dose RSV respectively. (D) Viral loads in the a-cellular fraction of the BAL of high dose RSV infected mice. (E,F) Total BAL cell and absolute neutrophil numbers per ml BAL at day 2 post infection in both isotype and anti-IL-17A treated mice. Data represent 3 mice per group for high dose RSV (10^7^ pfu/mouse) infected mice, and at least 5 mice per group for low dose RSV (10^6^ pfu/mouse) infected mice. (G) Representative scatter plots of CD11b and GR-1 surface stained BAL cells. * denote significance of p<0.05.

Local IL-17A production and airway neutrophilia occur simultaneously in mice, and correlate strongly in human patients. We next set out to establish a causal relationship between both phenomena. To confirm that local IL-17A enhances the early neutrophil infiltration in RSV-infected mice, we therefore neutralized IL-17A during the first days of infection using an IL-17A antibody. 6-week old BALB/c mice were given intra-peritoneal injections of 50 μg anti-IL-17A or isotype control antibody at both two days prior to intranasal infection and upon infection with RSV. Mice were infected with either a high dose RSV (1x10^7^ pfu), or a lower dose (1x10^6^ pfu) to detect subtle effects of the treatment. We monitored disease progression through daily body weight measurements and sacrificed mice at 48 hours post infection (Figure 5a, experimental design). We analyzed the number of neutrophils in BAL fluid by flow cytometry. 

High- but not lower-dose infected animals showed a modest decline in body weight, irrespective of antibody treatments (Figure 5 b,c). IL-17A neutralization did not affect the viral load in high dose infected mice (Figure 5d). Both total BAL cell (Figure 5e) neutrophil infiltration (Figure 5f) were more pronounced in high dose than in low dose infected mice (Figure 5f). The treatment with anti-IL-17A limited the infiltration of both cell populations in high-dose RSV infected mice (Figure 5e,f). We observed a similar trend for neutrophils, but no significance, in low-dose infected mice (Figure 5f,g representative scatterplots). 

We therefore conclude that early local IL-17A production is instrumental in the induction of innate neutrophil infiltration during primary RSV infection.

## Discussion

Elevated tracheal aspirate IL-17A levels in RSV patients were previously reported by Larranaga et al, Mukherjee et al, and Faber et al. [15,17,18]. The relevance of IL-17A presence for the innate phase of early pathology in patients, a hallmark of primary RSV infection in newborns, had however remained unclear. Our data show that local IL-17A levels are already elevated in the lungs of RSV-infected newborns early after ICU admission. The concentration of IL-17A in tracheal aspirate correlates strongly to neutrophil infiltration into the lung lumen. 

We demonstrated using primary RSV-infected mice that various lung intrinsic lymphocyte populations produce IL-17A during the innate immune response, of which CD4 T cells were a major contributor that increased in both absolute and relative numbers. Removal of IL-17A from the lungs using a depleting antibody significantly diminished airway neutrophil infiltration upon RSV infection *in vivo*. 

Local IL-17A production in RSV infection is biphasic [15] and may have a different impact throughout the disease. To assure we studied the innate phase of RSV disease, we selected patients with less than 7 days of disease history. As our patients have an average disease history of 4.2 days and a low standard deviation, we are confident that our samples were taken during the innate phase of the disease, despite not knowing the precise date that these patients were infected. 

Infection of Balb/c mice with a low dose RSV (1.0x10^6^ pfu) induced only limited neutrophil infiltration. Others previously reported an absence of local IL-17A in the airways of Balb/c mice when infected with this amount of RSV [13,35]. In line with these data, neutralization of IL-17A in low dose RSV infected mice did not significantly inhibit neutrophil infiltration, suggesting that high viral loads are required for early IL-17A production and subsequent neutrophil infiltration.

The identity of the CD4^+^ subtype of T cells that produces IL-17A during early primary RSV infection in BALB/c mice deserves further clarification. The promptness of IL-17A production by lung-resident CD4 T cells argues against involvement of V-D-J recombination of TCR genes and clonal T cell expansion (i.e., adaptive RSV-antigen driven response), that require several days to develop. We are not the first to report rapid IL-17A-producing cells in the lungs: so-called natural Th17 (nTh17) cells are an early source of IL-17A in lung inflammation [36]. Whereas conventional Th17 cells differentiate from peripheral naive CD4 T cells as a part of the adaptive response, nTh17 cells undergo a distinct thymic differentiation path and may perform bridging functions [37]. For these reasons we postulate that the CD4^+^ T cell population we observed in RSV-infected lungs may be nTh17 cells.

We also showed that IL-17A that is present during RSV infection strongly enhances the production of the neutrophil-attracting chemokine IL-8 by airway epithelial cells *in vitro* in a synergistic and virus replication-dependent fashion. Similar to our observations, others recently showed that recognition of viral replication intermediates, mimicked by transfection of poly-I:C, induces the expression of IL-8 in airway epithelial cells, which is synergistically enhanced by IL-17A [38]. IL-17A was described to augment the expression of KC through mRNA stabilization [39] in a TRAF5 dependent manner [40]. RSV induces IL-8 production independent from viral replication within 2 hours after infection [29]. Therefore the discrepancy we observed between the lack of enhancement of IL-8 mRNA, but the highly significant enhancement of IL-8 protein in combination with UV RSV may be related to our earlier timepoint of mRNA analysis. Nonetheless, IL-17A has a much greater synergistic effect in combination with live RSV, which suggests that RSV replication is highly relevant for potent induction of local IL-8 expression during infection. Higher viral loads have been correlated to RSV disease severity based on clinical scores [6]. Whether IL-17A and IL-8 contribute to this correlation remains to be investigated.

Based on our work and collective work by others, we anticipate that the role of IL-17A during primary RSV infection in infants may not be unique. RSV infects infants at a high frequency within the first year and may therefore reveal infant vulnerabilities where other diseases, e.g. influenza, do less so. Where a reduced functioning of the IL-12/IFN-gamma axis has been a preferred mechanistic explanation why newborns are particularly susceptible to pathogen infections, diminished functioning of this pathway function results in the increased deployment of the IL-23/IL-17A immune axis. Indeed, neonatal dendritic cells show an enhanced propensity to produce IL-23 [41], which is required for the activation of IL-17A production. Our findings here emphasize a pivotal role of IL-17A to newborn virus-induced airway pathology, and suggest IL-17A and its upstream inducers as candidate mediators to explore for intervention therapy and future clinical application.

## Supporting Information

Figure S1 **Human neutrophil gating strategy.** Representative scatter plots of human TA neutrophils stained for CD11b, CD14, and CD16.
(TIFF)

Figure S2 **Light-microscopy images of day 2 mock infected and RSV infected BAL cells.** Representative images of May-Grünwald&Giemsa stained BAL cells.
(TIFF)
