# Supplementary figures and images for: Local IL-17A Potentiates Early Neutrophil Recruitment to the Respiratory Tract during Severe RSV Infection
Source: PLoS One. 2013 Oct 23;8(10):e78461. doi: 10.1371/journal.pone.0078461 (PMC3806820; doi:10.1371/journal.pone.0078461)

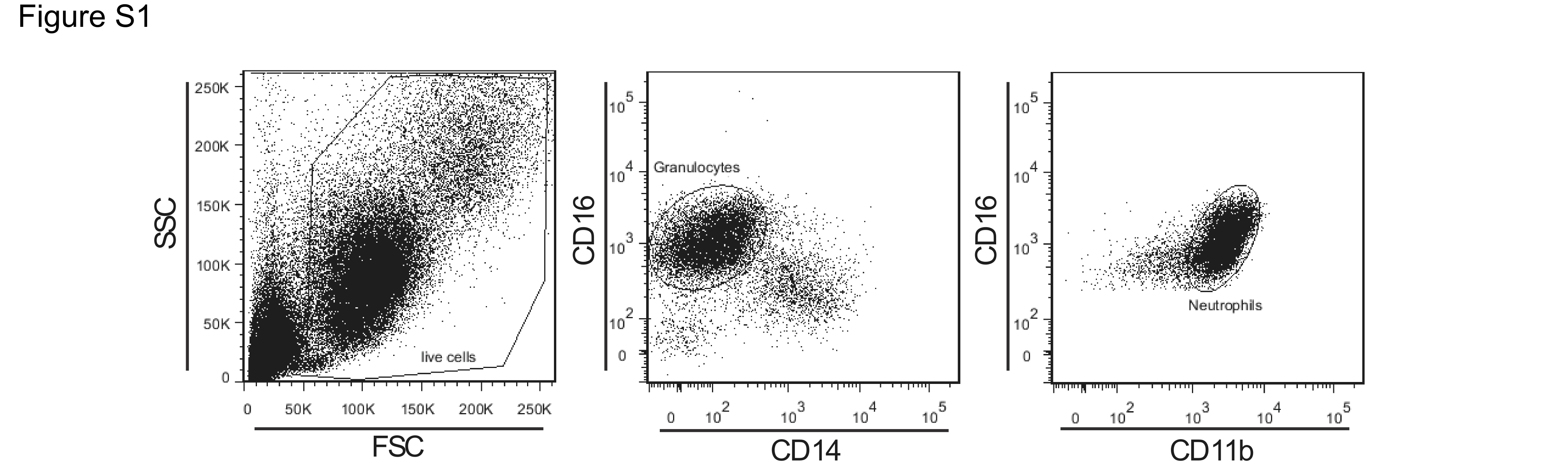

Supplement: Figure S1 — Human neutrophil gating strategy. Representative scatter plots of human TA neutrophils stained for CD11b, CD14, and CD16. (TIFF) [file pone.0078461.s001.tiff]

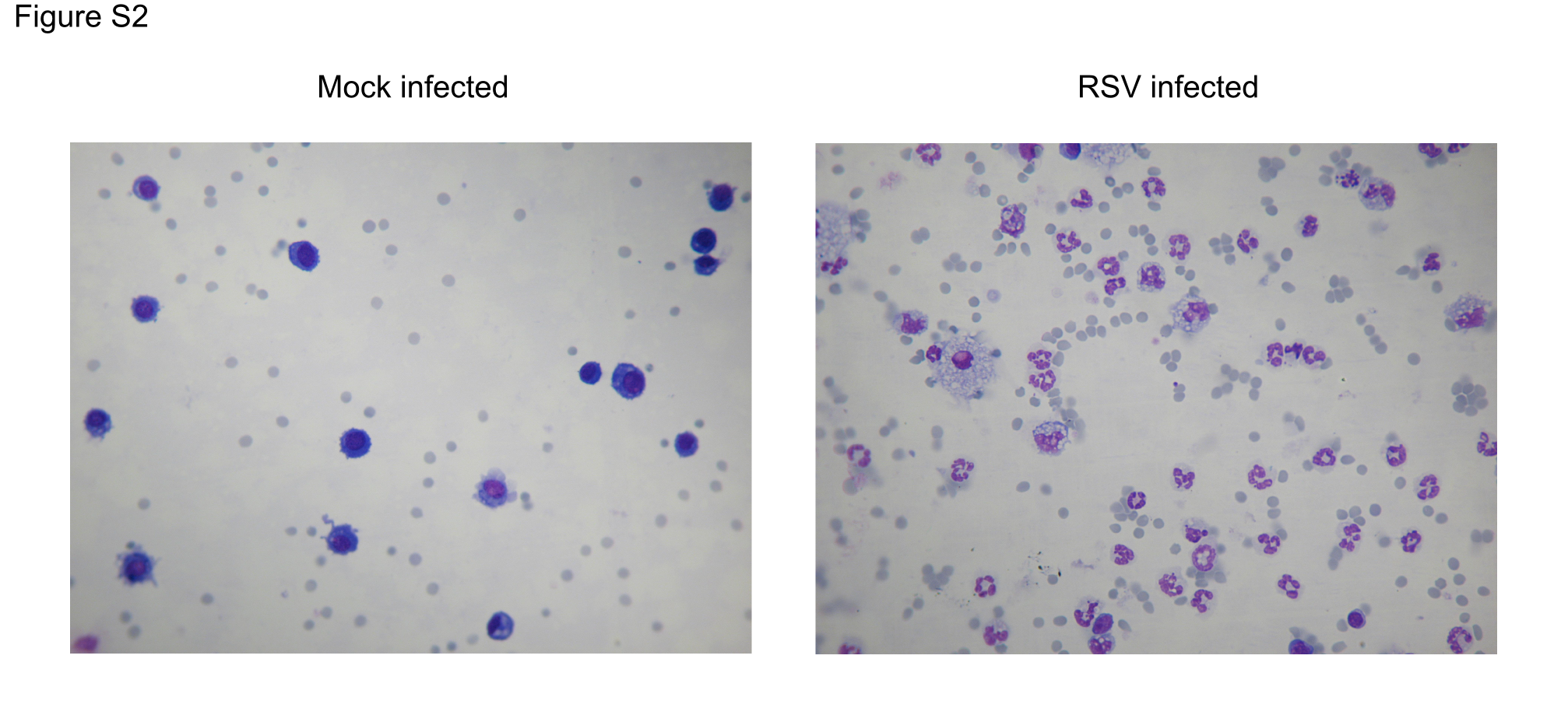

Supplement: Figure S2 — Light-microscopy images of day 2 mock infected and RSV infected BAL cells. Representative images of May-Grünwald&Giemsa stained BAL cells. (TIFF) [file pone.0078461.s002.tiff]
